# Schwannoma of the Great Auricular Nerve: A Rare Entity

**DOI:** 10.7759/cureus.90722

**Published:** 2025-08-22

**Authors:** Stella Fotiadou, Evgenia Daskalaki, Anastasios Stefanidis, Grigorios Panselinas, Anastasia Nikolaidou

**Affiliations:** 1 Department of Otolaryngology - Head and Neck Surgery, Theagenio Cancer Hospital, Thessaloniki, GRC; 2 Department of Pathology, Theagenio Cancer Hospital, Thessaloniki, GRC

**Keywords:** benign tumor, great auricular nerve, neck mass, schwannoma, surgical removal

## Abstract

Schwannoma of the great auricular nerve is an extremely rare benign tumor. We report the case of a 26-year-old woman who developed a slow-growing right lateral neck mass four years ago. Ultrasound scan and computed tomography were performed, and the mass was diagnosed as a cyst. Complete surgical removal was performed, and the histological report was consistent with a schwannoma. Postoperatively, the patient developed numbness of the ipsilateral auricle. Follow-up showed no recurrence, and the numbness improved. Although rare, schwannomas should be taken into account in the differential diagnosis of tumors located in the course of the nerves of the neck, and surgical treatment should be discussed with the patient.

## Introduction

Schwannoma, or neurilemmoma, is a benign tumor originating from Schwann cells, located in the peripheral nerve sheath. Schwann cells produce the myelin sheath that covers peripheral nerves [1]. Schwannoma is surrounded by a capsule. Its incidence is rare. The most common location of this tumor is extracranial in the head and neck, with an occurrence of 25-45% [1].

The cranial nerve that is mostly involved is the vestibulocochlear nerve. Schwannoma also arises from other cranial nerves, such as the facial nerve, glossopharyngeal nerve, vagus nerve, accessory nerve, and hypoglossal nerve. It can also arise from the cervical chain, sympathetic trunk, and branchial plexus [2]. It is usually present in the parapharyngeal space, while it may occur in the parotid gland and the submandibular gland. In the oral cavity, it can be seen in the tongue, palate, buccal mucosa, and lip. Orbit, ear, paranasal sinuses, larynx, and the subcutaneous layer of epidermis of the face are other sites of schwannoma [3].

Schwannoma is usually a benign tumor, but can rarely become malignant over time with a risk of less than 1%, transforming to epithelioid malignant peripheral nerve sheath tumor [1]. Clinically, a schwannoma is a unilateral tumor of the medial or lateral neck, a painless, mobile mass, firm in consistency, asymptomatic, with a slow growth over months [3]. In this case report, we present a rare schwannoma that originated from the great auricular nerve in the neck region.

## Case presentation

A 26-year-old woman was referred to the department of Otolaryngology- Head and Neck Surgery for evaluation of a neck mass. The patient presented with slow growth of a right lateral neck mass four years ago. The physical examination revealed a painless, mobile, solid mass, on the right neck area, 3 cm below the mandible. No palpable cervical lymph nodes were noted. The patient had no other problems in her medical history. 

**Figure 1:**
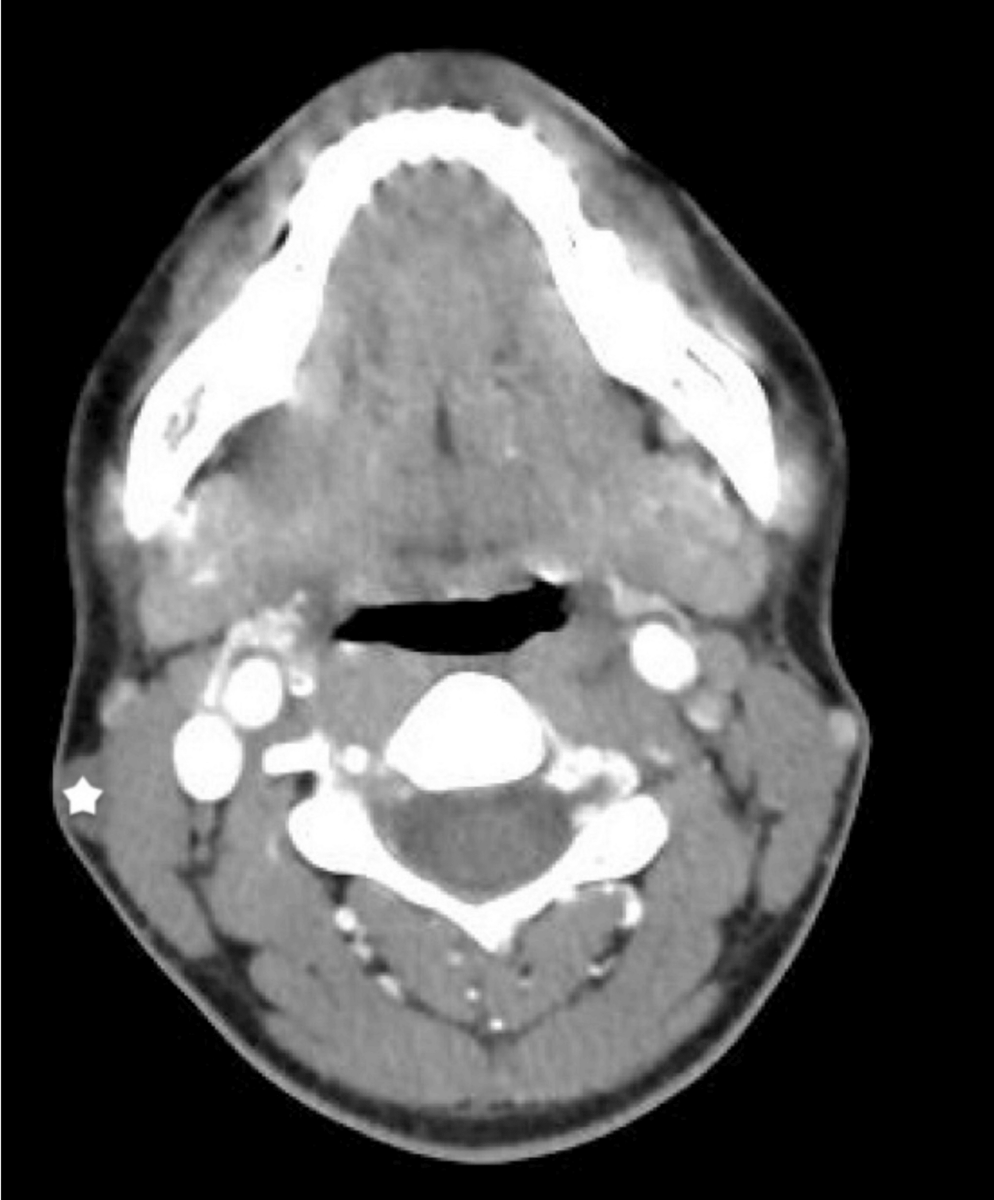
Contrast-enhanced CT scan of the neck showing an axial view of a neck mass, attached to the lateral aspect, middle third of the right sternocleidomastoid muscle (asterisk).

An ultrasound scan showed a mass beneath the right superior lobe of parotid gland, with a diameter of 1.02 x 0.46 cm, with subcutaneous location. The mass was hypoechoic with round boundaries and without vascular formations. It was possibly diagnosed as a sebaceous cyst. A contrast-enhanced CT scan of the neck showed a hypodense mass with a diameter of 0.8 x 1.2 x 2.1 cm in the right neck, attached to the lateral aspect, middle third of the sternocleidomastoid muscle (Figure 1). The mass was consistent with a cyst. 

**Figure 2:**
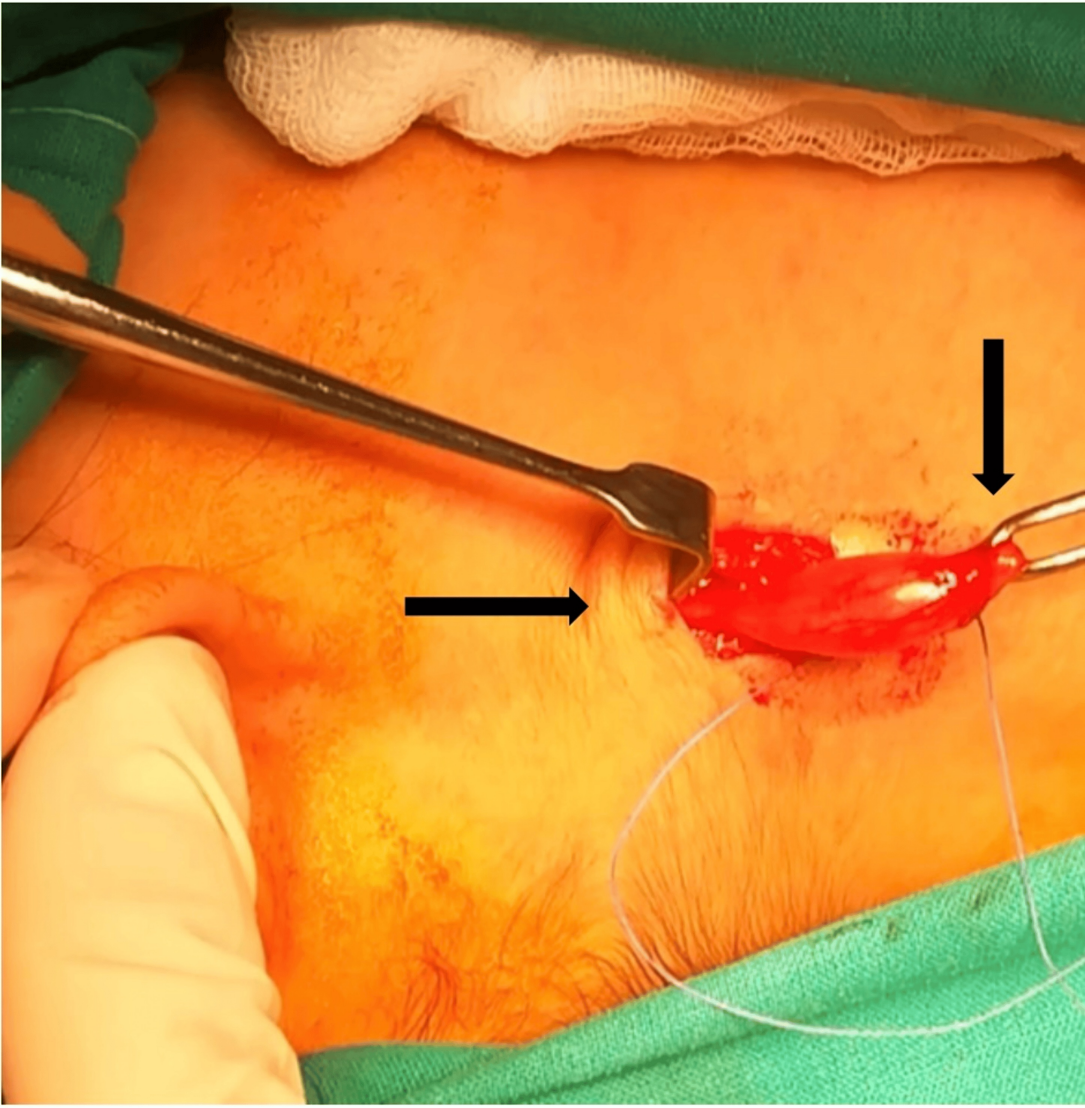
Surgical removal of a well-encapsulated mass of the great auricular nerve, with an upper and lower pole (arrows).

External excision was performed under general anesthesia. A small horizontal incision was performed 3 cm under the inferior part of mandible. During the operation, a solid whitish mobile mass was found. The mass was well-encapsulated and arrived from the great auricular nerve, through an upper and lower pole (Figure 2). The poles were ligated and the mass was dissected from the nerve sheath. The mass was completely removed. The skin was closed layer by layer. 

**Figure 3:**
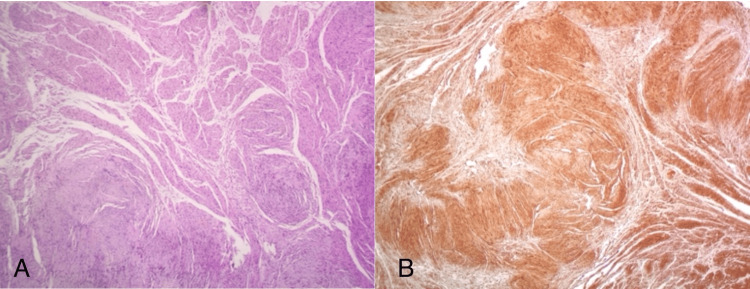
(A) Hematoxylin and eosin-stained section showing spindle cells without atypia with a storiform pattern. (B) The neoplastic cells presented a diffuse positivity for the immunohistochemical stain for the S-100 protein.

Histological examination showed a benign tumor with characteristics of schwannoma. The tumor consisted of spindle cells without atypia, with storiform pattern (Figure 3A). The neoplastic cells showed diffuse positivity for the immunostains for SOX-10 and S-100 protein (Figure 3B), while they were negative for Melan A, HMB45, pancytokeratin AE1-AE3, CD34, C-kit, desmin, caldesmon and SMA. The Ki67 proliferation index was positive to 2-3% of the tumor cells.

The patient was discharged the next day. Postoperatively, the patient complained about numbness of the ipsilateral auricle. The healing of the wound was excellent, and the sutures were removed on the 10th day. Upon follow-up after one month, the patient showed no recurrence, and the numbness of the periauricular area was improved.

## Discussion

Schwannoma is a benign tumor formed by Schwann cells. It is rare, and one arising from the great auricular nerve is even rarer. A few cases have described a great auricular nerve schwannoma in the English literature [4]. Schwannoma of the great auricular nerve has mostly been reported in Asia, mainly in China, Japan, and Korea [5,6]. There is only one reported case in the Western world, in the United Kingdom [4]. This is the second reported case of schwannoma of the great auricular nerve in a Western population. 

Clinically, it usually appears as a slow-enlarging, solid, painless mass. As it grows, it may cause pain in the periauricular region or sensory dysfunctions in the ear lobe [5]. In most cases, it remains asymptomatic for years [4]. In the report from the United Kingdom cases, the patient presented with a painless lateral neck mass [4], as in our case. In Asian cases, patients presented with a painless neck or postauricular mass [6].

The great auricular nerve is a pure sensory nerve from the C2 and C3 cervical nerves, branches of the cervical plexus [4]. It penetrates the middle part of the sternocleidomastoid muscle and divides into anterior and posterior branches. The posterior branch forms a loop around the sternocleidomastoid muscle and supplies the skin over the mastoid process and postauricular region [5]. It is responsible for the sensation of the ear lobe, lower pinna, and periauricular region [5].

Diagnostic imaging is not always helpful in determining a schwannoma. CT reveals a benign mass with rounded boundaries and internal vascularity [4]. In the case reported by Oh et al., from the United Kingdom, CT showed a neck mass, without specific characteristics of schwannoma [4]. MRI is more specific for the determination of the schwannoma and tends to show inhomogeneous intermediate signal on T2 and intermediate signal on T1. Its shape is oval with well-defined boundaries [6]. In some cases, in Asia, due to clinical suspicion of neurilemmoma, MRI was conducted [6]. On ultrasonography, a schwannoma might appear as a subcutaneous hypoechoic cyst [5]. 

The gold standard treatment of schwannoma is complete surgical removal [7]. Incomplete excision can lead to recurrence. Enucleation of the tumor has no better outcomes [8]. The asymptomatic, slow growth of schwannoma justifies the observational approach, as well; however, as the size increases, symptoms often appear due to the mass pressing on the adjutant structures, resulting in neurologic deficit [3,5]. In rare cases, schwannomas become malignant [1]. Extracranial schwannomas of the head and neck are considered radio-resistant [3]. So, the surgical excision of schwannomas is the treatment of choice. 

Histologically, there are specific characteristics of a schwannoma. It is surrounded by a well-formed capsule and consists of Antoni A and Antoni B areas and Verocay bodies. Antoni A areas have plenty of spindle cells with round nuclei. The cells have no atypia or necrosis. Antoni B areas have fewer spindle cells within a myxoid matrix. Verocay bodies consist of two lines of nuclei and cells with an elliptical arrangement. Protein S-100 expression is positively stained in the case of schwannomas [9]. 

Postoperatively, there is numbness in the periauricular region [10]. The complete surgical excision of the schwannoma and the enucleation with preservation of the nerve lead to the same result [10,11]. When a schwannoma is not suspected preoperatively, numbness of the preauricular area leads to possible discomfort for the patient [10]. Postoperative complications should be discussed with the patient. In some cases, there was improvement of the numbness [11].

In our case, the schwannoma originated from the great auricular nerve, a rare entity. It is the second reported case in the West and the first reported case in Southern Europe. The patient had no pain. The mass was growing slowly for months. CT imaging did not indicate a schwannoma. It was completely surgically removed and had the histological characteristics of a schwannoma. Postoperatively, there was periauricular numbness. After months, the numbness had improved, and the patient was satisfied.

## Conclusions

Schwannomas are rare benign neoplasms. Their origin from the great auricular nerve is even rarer. They appear clinically as slow-growing masses. Tumors in the neck, located in the course of nerves, must be suspected as schwannomas. Imaging with CT/MRI could be helpful in the differential diagnosis. Histological evaluation establishes the diagnosis. The surgical treatment and postoperative complications should be thoroughly discussed with the patient.

## References

[1] Loperfido A, Celebrini A, Fionda B, Bellocchi G, Cristalli G (2023). Diagnostic and therapeutic strategy for vagal schwannoma: case series and literature review. Medicina (Kaunas).

[2] Phulware RH, Sardana R, Chauhan DS, Ahuja A, Bhardwaj M (2022). Extracranial schwannomas of the head and neck: a literature review and audit of diagnosed cases over a period of eight years. Head Neck Pathol.

[3] Sharma P, Zaheer S, Goyal S, Ahluwalia C, Goyal A, Bhuyan G, Mandal AK (2019). Clinicopathological analysis of extracranial head and neck schwannoma: a case series. J Cancer Res Ther.

[4] Oh S, Abou-Foul A, Patel S, Wilson P (2021). Great auricular nerve schwannoma: a rare presentation and literature review. BMJ Case Rep.

[5] Kim KS, Lee H, Choi JH, Hwang JH, Lee SY (2020). Schwannoma of the posterior branch of the great auricular nerve. Arch Craniofac Surg.

[6] Xu C, Sun Q (2019). Great auricular nerve schwannoma in neck region: a case report with the risk of medical disputes. BMC Neurol.

[7] Minovi A, Basten O, Hunter B, Draf W, Bockmühl U (2007). Malignant peripheral nerve sheath tumors of the head and neck: management of 10 cases and literature review. Head Neck.

[8] Ohnishi YI, Nakajima N, Fujiwara S, Moriwaki T, Arita H, Kishima H (2020). A sufficient surgical window for deep-seated extracranial schwannomas in the craniocervical junction by the anterolateral approach. Neurospine.

[9] Vrinceanu D, Dumitru M, Popa-Cherecheanu M, Marinescu AN, Patrascu OM, Bobirca F (2023). Extracranial facial nerve schwannoma-histological surprise or therapeutic planning?. Medicina (Kaunas).

[10] Yan F, Desiato VM, Nguyen SA, Lentsch EJ (2021). Impact of greater auricular nerve sacrifice during parotidectomy on quality of life. Head Neck.

[11] Min HJ, Lee HS, Lee YS (2007). Is it necessary to preserve the posterior branch of the great auricular nerve in parotidectomy?. Otolaryngol Head Neck Surg.

